# A Manual of Venereal and Sexual Diseases

**Published:** 1902-03

**Authors:** 


A Manual of Venereal and Sexual Diseases. By Wm. A. Hackett, Ph.G., M. C. P. S. (Ont.), Professor of Dermatology and Venereal Diseases, Michigan College of Medicine and Surgery; and N. E. Aronstane, M. D., Ph G., Assistant in Chemistry and Clinical Dermatology in the same institution. Chicago: G. P. Engelhard & Co. 1901.

This small volume, 203 pages, is divided into four parts in which the authors deal with (1) gonorrhea and its complications, including circumcision, (2) chancroids and their complications. (3) syphilis and (4) sexual psychoses. Although too much cannot be expected from a work of this size, nevertheless many things that are today considered as up-to-date practice are entirely omitted.

Objections must be taken to the advice that a catheter is necessary either in the treatment of anterior or posterior urethritis. In these conditions medication can be made without its use; and thus fully 50 per cent. of irritation avoided. Mention should have been made of leeches in acute prostatitis, for with this mode of treatment all symptoms are obliterated and the glands brought to an apparently normal condition in a few hours, instead of days, as must be the case under the advised treatment.

More space should have been given the symptoms of cystitis, so as to differentiate it from posterior urethritis.

The treatment advised for cystitis seems to be that in vogue years ago. Today it is recognised that a patient with acute gonorrheal cystitis need not go to bed, but all distressing symptoms may be relieved and a complete cure obtained in a few hours.

In mentioning the complications of stricture the most common one—namely, infiltration, has been omitted. The authors say under treatment, "treat the urethritis before touching the stricture;" this is the soundest rule that could be advanced and one that if followed would be derivative of much better results than is generally obtained. When speaking of retention no mention is made of local anesthesia to the strictured tissue. Considering that the lumen of the canal is never closed, that the apparent closure is due to spasmodic contraction which is increased the moment an instrument touches it, the result can be imagined where cocaine or eucaine is used.

Syphilis is well considered for the space given it, yet there are a few points with which the writers may be differed from, chief among which is the recognised fact that, except in the presence of extragenital chancre (and there should be good ground for the diagnosis here) treatment should not be commenced until the appearance of the secondaries.

In Part IV. the writers should have differentiated between masturbation and onanism, as they are two entirely distinct habits, the former not usually acquired until marriage, whereas the latter begins at puberty; then, again, the treatment is entirely different in each case. In the treatment of these psychoses mention should have been made of the mixture known as arsenauro, as the result obtained from its use is marvelous in these conditions. J. H. D.

